# Supplementary material for: Free Fatty Acids Signature in Human Intestinal Disorders: Significant Association between Butyric Acid and Celiac Disease
Source: Nutrients. 2021 Feb 26;13(3):742. doi: 10.3390/nu13030742 (PMC7996737; doi:10.3390/nu13030742)
Supplement: Supplementary file 1 [file nutrients-13-00742-s001.pdf]

Title: Free fatty acids signature in human intestinal disorders: significant association between butyric acid and celiac disease

Simone Baldi[1], Marta Menicatti[4], Giulia Nannini[1], Elena Niccolai[1], Edda Russo[1], Federica Ricci[1], Marco Pallecchi[4], Francesca Romano[5], Matteo Pedone[2], Giovanni Poli[2], Daniela Renzi[3], Antonio Taddei[1], Antonino S. Calabrò[3], Francesco C. Stingo [2], Gianluca Bartolucci[4], Amedeo Amedei[1,6]

1 Department of Clinical and Experimental Medicine, University of Florence, Florence 50134, Italy

2 Department of Statistics, Computer Science, Applications "G. Parenti", Florence 50134, Italy

3 Department of Biomedical, Experimental and Clinical Sciences "Mario Serio" University of Florence, Florence 50134, Italy

4 Department of Neurosciences, Psychology, Drug Research and Child Health Section of Pharmaceutical and Nutraceutical Sciences University of Florence, Florence 50139, Italy

5 Laboratory of Clinical Pathology, Versilia Hospital, Lido di Camaiore 55041, Italy

6 SOD of Interdisciplinary Internal Medicine, Azienda Ospedaliera Universitaria Careggi (AOUC), 50134 Florence, Italy.

Corresponding author: Prof. Amedeo Amedei, Department of Experimental and Clinical Medicine, University of Florence, Largo Brambilla 3, Florence 50134, Italy. aamedei@unifi.it Telephone/ Fax: +39-55-2758330

Supporting information

S1. Instrumental and chemicals

Methanol and tert-butyl methyl ether (Chromasolv grade), sodium bicarbonate, sodium chloride and hydrochloric acid (Reagent grade), [$^2H_3$]Acetic, [$^2H_5$]Propionic, [$^2H_7$]iso-Butyric and [$^2H_9$]iso-Valeric (used as internal standards or ISTDs), acetic, propionic, butyric, iso-butyric, valeric, iso-valeric, 2-Methylbutyric, hexanoic, heptanoic, octanoic, and nonanoic acids (analytical standards grade) were purchased by Sigma-

Aldrich (Milan, Italy). MilliQ water 18 MΩ cm was obtained from Millipore's Simplicity system (Milan - Italy).

S2. GC-MS method

The FFAs in the samples were analyzed as free acid form using an Agilent J&W DB-FFAP column 30 m length, 0.25 mm internal diameter and 0.25 m of film thickness by using the oven temperatures' program, as follows: initial temperature of 50 °C for 1 min, then it was increased to 150 °C at 30 °C/min, finally grow up to 250 °C at 20 °C/min was held for 6.67 min. A 1 µL aliquot of extracted sample was injected in splitless mode (splitless time 1 min) at 250°C, while the transfer line temperature was 280°C. The used carrier gas was helium and its flow rate maintained at 1 mL/min for the whole run time. The MS acquisition was carried out in single ion monitoring (SIM) by apply a proper dwell time (20 ms for each ion monitored) to guarantee an acquisition frequency of 4 cycle/s.

The quantitative determination of FFAs in each sample was carried out by the ratio between the area abundance of the analytes and the area abundance of respective labeled internal standard (isotopic dilution method). The value of this ratio was named Peak Area Ratio (PAR) and it was used as abundance of each analyte in the quantitative evaluation. The ionic FFAs' signals and the reference internal standards used for the quantitation of each FFAs were reported in the **Table S1**.

**FFA**	**Rt (min.)**	**Quan. Ion (m/z)**	**Qual. Ion (m/z)**	**ISTD [Quan. ion (m/z)]**
Acetic acid	4.95	60	-	$[^2H_3]$Acetic [63]
Propionic acid	5.37	74	73	$[^2H_5]$Propionic [77]
iso-Butyric acid	5.49	73	88	$[^2H_7]$iso-Butyric [77]
Butyric acid	5.78	60	73	$[^2H_5]$Propionic [77]
iso-Valeric acid	5.97	60	73	$[^2H_9]$iso-Valeric [63]
2-Methylbutyric acid	5.98	74	73	$[^2H_9]$iso-Valeric [63]
Valeric acid	6.29	60	73	$[^2H_9]$iso-Valeric [63]
Hexanoic acid	6.80	60	73	$[^2H_9]$iso-Valeric [63]
Heptanoic acid	7.29	60	73	$[^2H_9]$iso-Valeric [63]
Optanoic acid	7.80	60	73	$[^2H_9]$iso-Valeric [63]
Nonanoic acid	8.27	60	73	$[^2H_9]$iso-Valeric [63]
Decanoic acid	8.75	60	73	$[^2H_9]$iso-Valeric [63]
Dodecanoic acid	9.64	60	73	$[^2H_9]$iso-Valeric [63]
Tetradecanoic acid	10.67	60	73	$[^2H_9]$iso-Valeric [63]
Hexadecanoic acid	12.11	60	73	$[^2H_9]$iso-Valeric [63]
Octadecanoic acid	14.19	60	73	$[^2H_9]$iso-Valeric [63]

Table S1. Retention times (Rt) and ionic signals used for quali-quantitation of FFAs and relative ISTD used.

The GC-MS profiles from the samples showed five unknown peaks (Rt 8.75, 9.64, 10.67, 12.11 and 14.19 min respectively), with the characteristic fragmentation of carboxylic acids. By considering the selectivity factor of the chromatographic system used toward the carboxylic acids and the relative abundances of the characteristic fragmented ions, these unknown peaks could be as follows:

- decanoic acid (Rt=8.75 min.);
- dodecanoic acid (Rt=9.64 min.);
- tetradecanoic acid (Rt=10.67 min.);
- hexadecanoic acid (Rt=12.11 min.);
- octadecanoic acid (Rt=14.19 min.);

S2.1 Standard solutions and calibration levels

The stock solutions of each analyte and each ISTD were prepared in mQ water at 50 mg/ mL^{-1} and stored at 4 °C.

Since the quantity of each FFA in the samples could be different, distinct concentration ranges of each analyte were defined. Therefore, to easily build up these calibration levels, two working mixtures of analytes (Mix 1 and Mix 2) and a mixture of ISTDs in 10 mM $NaHCO_3$ solution were prepared. The compositions and the concentrations of these mixtures are reported in the **Table S2**.

Acids Mixtures	Mix 1 (µg/mL)	Mix2 (µg/mL)	**ISTDs**	ISTDs mixture **(µg/mL)**
Acetic acid	1000	100	[$^2H_3$]Acetic	1000
Propionic acid	100	10	[$^2H_5$]Propionic	100
Butyric acid	100	10		
iso-Butyric acid	100	10	[$^2H_7$]iso-Butyric	100
iso-Valeric acid	100	10	[$^2H_9$]iso-Valeric	100
2-Methylbutyric acid	100	10		
Valeric acid	100	10		
Hexanoic acid	100	10		
Heptanoic acid	100	10		
Octanoic acid	100	10		
Nonanoic acid	100	10		

Table S2. Compositions and concentrations of standard and ISTDs mixtures.

A five levels calibration curve was prepared by adding proper volumes of Mix 1 or 2 solution, 10 µL of ISTDs mixture, 0.1 mL of tert-butyl methyl ether and 20 µL of 6 M HCl + 45 mM NaCl solution in 0.5 mL centrifuge

tube. Then, each tube was stirred in vortex apparatus for 2 minutes, centrifuged at 10000 rpm for 5 minutes, and finally the solvent layer was transferred in vial with microvolume insert and analyzed three times by GC-MS method. Final concentrations of calibration levels are 2.5, 5.0, 10.0,25.0 and 50.0 µg/mL for Acetic acid, while the other acids are 0.25, 0.50, 1.00, 2.50 and 5.00 µg/mL.

S2.2 Calibration curves

Calibration curves of analytes were obtained by plotting the peak area ratios (PAR), between quantitation ions of each analyte and relative ISTD, versus the nominal concentration of the calibration solution. A linear regression analysis was applied to obtain the best fitting function between the calibration points.

In order to obtain reliable limit of detection (LOD) and limit of quantitation (LOQ) values, the standard deviation of response and slope approach was employed. The estimated standard deviations of responses of each analyte were obtained by the calculated standard deviation of y-intercepts (SDY-I) of regression lines.

The obtained linear regressions coefficients, the r-squared (R2) and the estimated LOD values for each analyte are reported in the **Table S3**.

**Compound**	**Slope** (PAR/µg mL^{-1})	**Intercept** (PAR)	**R^2**	**LOD SDY-I** (µg mL^{-1})
Acetic acid	0.079	+0.017	0.999	0.6
Propionic acid	0.942	-0.009	0.999	0.1
Butyric acid	3.039	+0.184	0.999	0.1
isoButyric acid	1.124	+0.020	0.999	0.1
isoValeric acid	1.005	-0.010	0.999	0.1
2-Methylbutyric acid	0.980	-0.001	0.999	0.1
Valeric acid	0.912	-0.030	0.999	0.1
Hexanoic acid	1.062	-0.018	0.999	0.1
Heptanoic acid	0.959	-0.028	0.999	0.1
Optanoic acid	0.824	+0.020	0.999	0.2
Nonanoic acid	0.578	+0.119	0.999	0.1

Table S3. Linear regressions data, R-squared and LOD values obtained for each analyte.

Concerning the quantitative evaluation of the acids not included in Table S3, it was use the calibration parameters obtained from the nonanoic acid.
